# PV cells and modules – State of the art, limits and trends

**DOI:** 10.1016/j.heliyon.2020.e05666

**Published:** 2020-12-15

**Authors:** Vítězslav Benda, Ladislava Černá

**Affiliations:** Czech Technical University in Prague, Faculty of Electrical Engineering, Department of Electrotechnology, Technická 2, 166 27 Praha 6, Czech Republic

**Keywords:** Electrical engineering, Energy, Environmental science, Photovoltaics, PV technology, Crystalline silicon PV modules, Thin film PV modules, PV module service life, PV module price, Levelized cost of energy

## Abstract

The key components of photovoltaic (PV) systems are PV modules representing basic devices, which are able to operate durably in outdoor conditions. PV modules can be manufactured using different materials by different fabrication technologies. The main criteria supporting or limiting a successful placement of particular technologies on the market is the cost of electricity produced by PV systems. The Levelized Cost of Energy (LCOE) method takes into account the investment cost, the operating costs, and the total energy produced during the system service life. The influence of price, efficiency and service life of PV modules on LCOE (together with the availability of materials) sets limits for applicable technologies.

Over the past 15 years a categorisation of generations of PV cell and module technology groups has been frequently used. The main features of individual technology groups are discussed from the view of the above criteria. Currently, PV modules are required to have: efficiency higher than 14%, price below 0.4 USD/W_p_ and service life of more than 15 years. At present, the wafer-based crystalline silicon technologies have best met the criteria due to their high efficiency, low cost and long service time; and due to the abundance of materials, they are set to lead in future PV power generation.

## 1 Introduction

**Figure 1:**
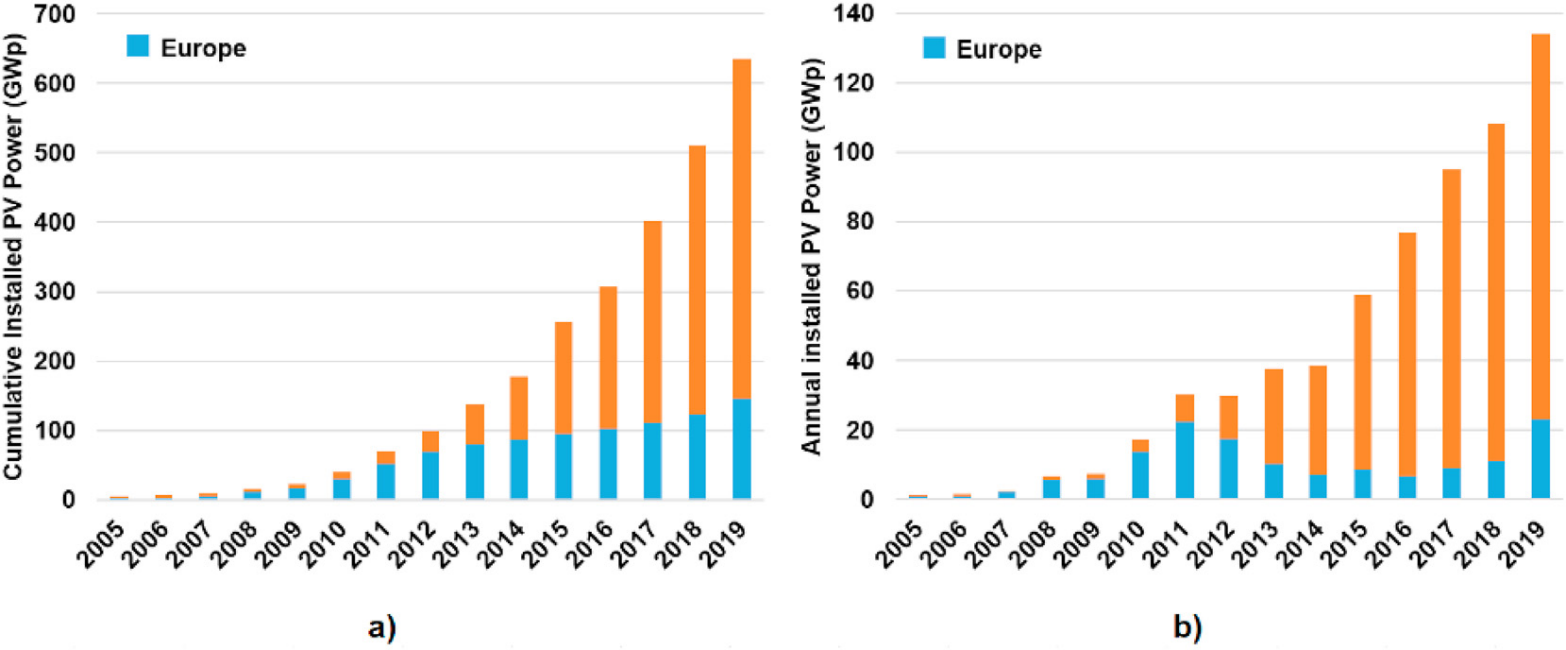
Development in photovoltaics: a) Global cumulative installed PV power in period 2007–2019. b) Global annual installed PV power in period 2007–2019.

Photovoltaics is currently one of the world's fastest growing energy segments. Over the past 20 years advances in technology have led to an impressive reduction in the cost of photovoltaic modules and other components, increasing efficiency and significantly improving both the reliability and yield of the system, resulting in reduced electricity prices. This is associated with the rapid growth in installed capacity of photovoltaic power plants. The cumulative PV capacity installed worldwide exceeded 635 GW_p_ in 2019 [1], of which over 130 GW_p_ was installed within the year. The development of the cumulative capacity is shown in Figure 1a. As shown in Figure 1a, the cumulative installed capacity increased thirty times between 2009 and 2019. Annual production (installed capacity) increased over the same period fifteen times to 130 GW_p_, as shown in Figure 1b and, in 2019, photovoltaics constituted more than 45% of new global electricity generation capacity additions.

In the past, the development of the photovoltaic industry was dependent on subsidies but the current rapid development is possible only when the price of electricity produced by PV systems is already low enough to compete with the price of electricity produced by other means. This paper discusses the influence of price, efficiency and service life of PV modules on LCOE (along with the availability of materials) and the resulting limits for the relevant technologies.

## 2 The price of electricity produced by photovoltaic systems

The price of electricity produced by a system (e.g. photovoltaic) is usually determined by a system levelized cost of energy analysis (LCOE) which allows different production methods to be compared [2].

If the system service life is *n* years, the price of energy can be expressed in simplified form as the sum of the total cost of acquisition and operation of the system (investment costs *IC*_*k*_, including interest and inflation in individual years and operating costs *OC*_*k*_ in the *k*^*th*^ year of operation) and the energy produced in each year. Since the time of realization of photovoltaic systems does not usually exceed one year, the LCOE can be expressed in a simpler form

(1) $$LCOE=\;\frac{\sum_{1}^{n}IC_{k\;}+\sum_{1}^{n}OC_{k}}{\sum_{1}^{n}EP_{k}}\;\approx \frac{IC+nOC_{AV}}{EP},$$

where *IC* is the total purchase price, *OC*_*AV*_ is the average annual operating cost, *n* is the service life of the system (in years) and *EP* is the total energy produced by the equipment over its service life.

Degradation processes associated with the operating environment occur in photovoltaic modules and their efficiency decreases in time. If the service life *n* is taken as the number of years to reduce the efficiency of the modules to 80% of the initial value, the LCOE calculation will be simplified (with linear decrease in efficiency with time and neglecting fluctuations of solar radiation in each year) and the price of electricity produced can be approximated [3].

(2) $$LCOE\approx \frac{IC\left(1+\frac{OC_{AV}}{IC}n\right)}{EP_{1}\times 0,9n}=\frac{IC}{EP_{1}}f\left(n;\varepsilon \right),$$

where ε *= OC*_*AV*_*/IC* and *EP*_*1*_ is the energy produced for the first year of operation. It depends on the module area *A*_*m*_, yearly irradiation on the module surface *H*_*sy*_, converter efficiency *η*_*c*_, and module efficiency *η*_*m*_ as

(3) $$EP_{1}=\;A_{m}H_{sy}\eta_{c}\eta_{m}.$$

Then, the price of electricity produced can be approximated as

(4) $$LCOE\approx \frac{IC}{A_{m}H_{sy}\eta_{c}\eta_{m}}f\left(n;\varepsilon \right).$$

**Figure 2:**
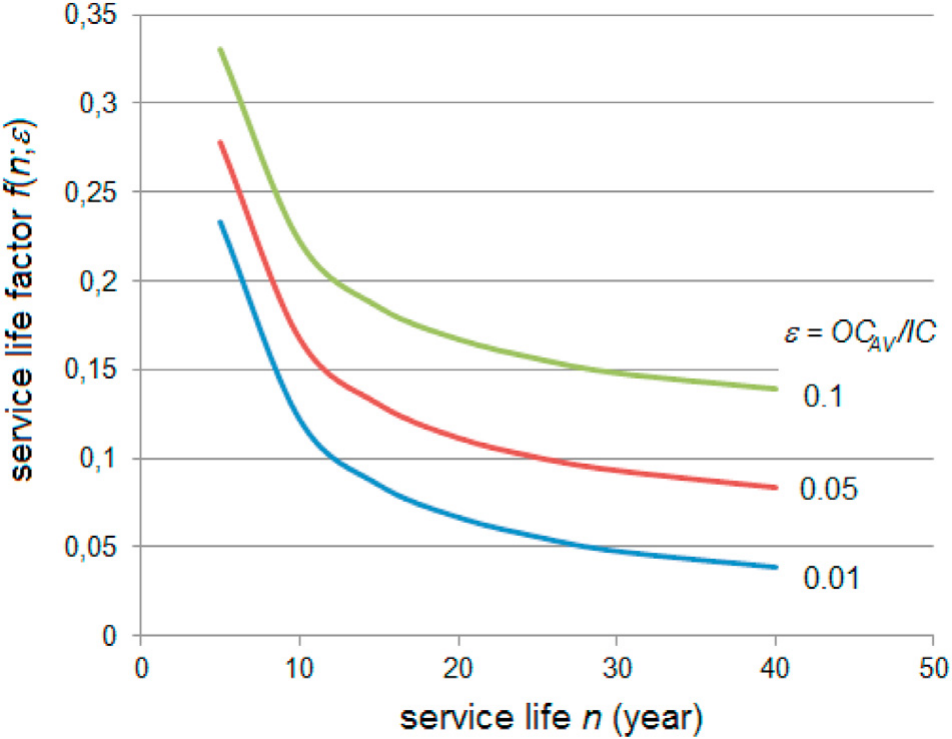
The service life factor *f(n;ε)* in dependence on the service life *n*.

The dependence of the function *f(n;ε)* on the service life *n* is shown in Figure 2. Obviously, with a shorter system service life (less than 15 years), LCOE increases rapidly with the same ratio of investment cost and module efficiency. Thus, the modules' service life of for energy generation should be longer than 15 years, which leads to considerations of module operation reliability. A shorter service life would be acceptable only in the case of extremely low investment costs to keep the product *IC.f(n;ε)* acceptably low.

**Figure 3:**
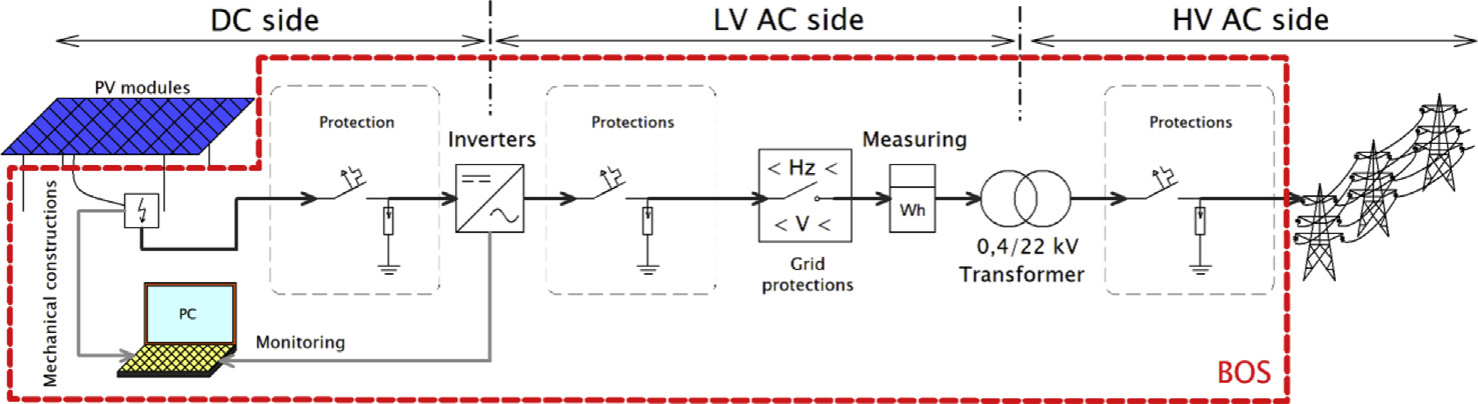
A simplified scheme of the PV system.

Attention is usually paid to achieving a minimum purchase price for the system and the generated energy. A simplified scheme of the PV system is shown in Figure 3. The photovoltaic system is usually divided into photovoltaic modules and other BOS (balance of system) components, which is a legacy from the time when photovoltaic modules accounted for the largest part of the cost of a photovoltaic power plant.

Although the module price is given as the price per unit of installed nominal power, the area required to generate the specified power depends on the efficiency of the modules. With the space requirement per unit of power, the required support surface area and the length of the cabling are part of the BOS price. The efficiency of a photovoltaic generator plays a significant role in the cost of a part of the BOS (the price of inverters, transformers, protections and computer technology does not depend on the PV system area). The efficiency of the modules is important in terms of optimizing the cost of the PV system. As module efficiency increases, the cost of BOSs decreases, while the cost of modules increases. However, if these system components costs are comparable on the market, there are also the so-called soft costs, which represent expenses connected with project, land, administration, margins, taxes, etc. These may vary with the location of the installation and the type of installation, and their share in the total price becomes significant.

**Figure 4:**
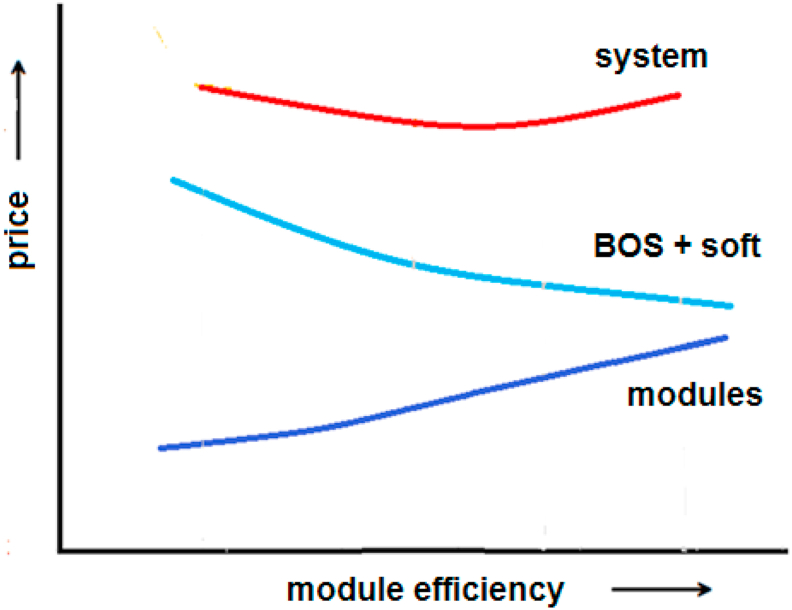
Module efficiency influence on PV system price.

The general influence of module efficiency on the price of the PV system parts and the PV system is schematically shown in Figure 4. According to Eq. (4), LCOE (at a constant investment cost and service life factor) decreases with increasing module efficiency.

Improved module efficiency can be achieved by improving design and technology such as reducing optical losses (reducing reflectivity, reducing surface shaded contacts), reducing recombination losses (increasing material quality, reducing surface recombination) and reducing electrical losses (busbar optimization, reducing contact resistance). A larger number of technological operations and higher-quality production equipment are then needed for manufacture. This is reflected in the price increase of PV cell technology. There is a limit for the additional cell production costs to get the same LCOE. For crystalline silicon an increase of 1% in cell efficiency would require the increase of cell production cost to be less than 25% for the process to be accepted [4, 5].

**Figure 5:**
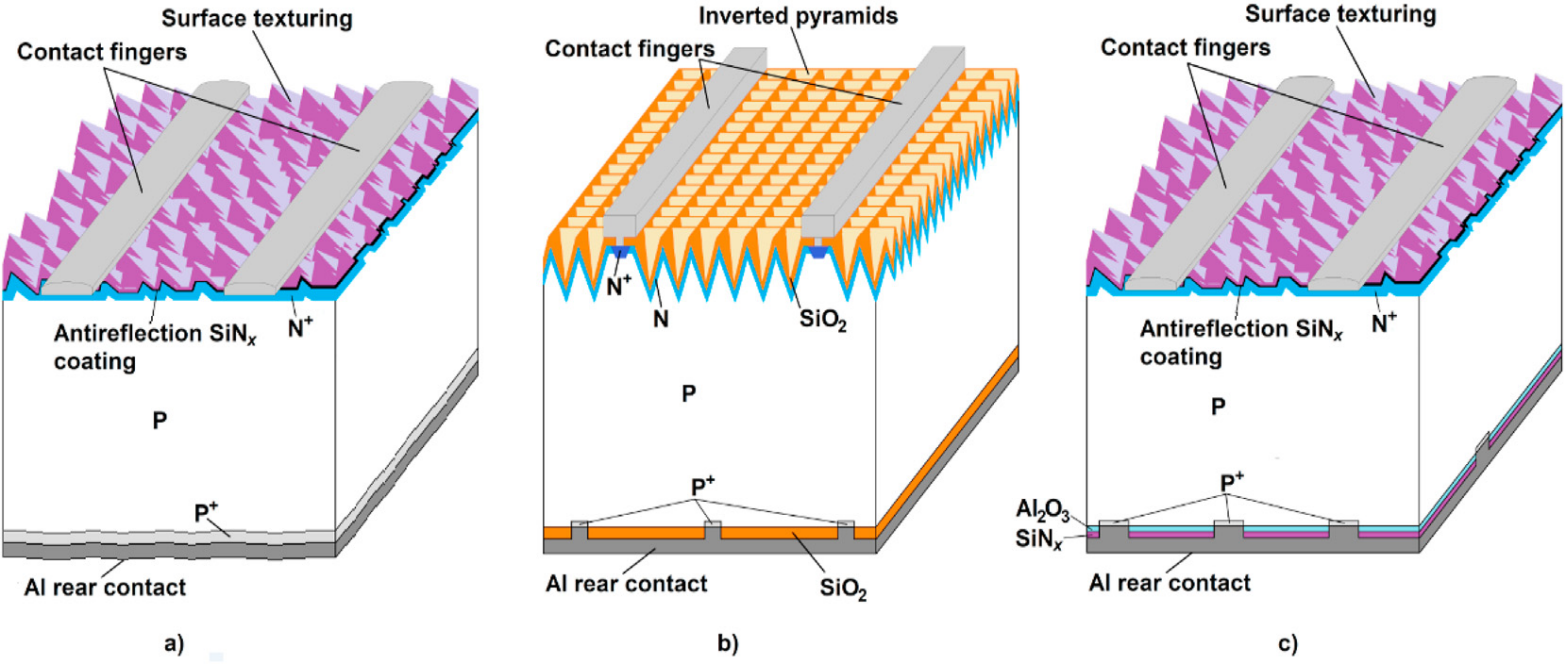
Development in crystalline silicon cell structures [36] a) A standard BSF crystalline silicon cell structure. b) The PERC structure as developed in 1988 (modified after [6]). c) The present PERC structure (modified after [8]).

As an example, the development in crystalline silicon cells may be taken. During their development in the 1980's, the BSF (Back Surface Field) technology allowing cell fabrication from starting P-type material without expensive photolithography and vacuum depositions was introduced in mass fabrication. The standard cell (BSF) structure is shown in Figure 5a. The principle of increasing the cell efficiency by decreasing surface recombination rate by covering the surface with a suitable dielectric layer has been known since the 1980's and was demonstrated by preparing the PERC (Passivated Emitter Rear Contact) cells with 23% efficiency in 1988 [6, 7] using float zone starting material and several photolithographic processes (the cell structure is shown in Figure 5b). However, at that time the cost difference between the BSF technology and the PERC technology was so high that it was not accepted for mass production and the BSF cell efficiency was mostly improved by improving the bulk material quality and front side optimisation (surface texturing, antireflection and passivating SiN_x_:H layer, contact pattern optimisation) to about 19%.

At the same time, mass production allowed the reduction of material costs and optimization of the use of production equipment. The next efficiency increase was possible by decreasing rear contact recombination using PERC technology. Development of Al_2_O_3_ passivation layer deposition and replacement photolithography by local laser ablation decreased the technology cost increment connected with three additional technological steps (rear surface etching, passivating Al_2_O_3_ layer combined with SiN_x_:H layer deposition and local windows opening by laser ablation) to an acceptable level. The present PERC cell structure is demonstrated in Figure 5c. The PERC technology took its position in mass production in 2016 [9] and gave an efficiency increase to 20%–22% with an acceptably low increase of the fabrication cost. PERC cells are quickly becoming the new standard and it is expected to become the leading technology in the near future [10]. Although N-type based crystalline silicon technologies - IBC (Interdigitated Back Contact), HJT (HeteroJunction Technology), PERT (Passivated Emitter Rear Totally diffused) and TOPcon (Tunnel Oxide Passivated Contact) - have higher efficiencies (23%–25%) and lower temperature coefficient in comparison with P-type based technologies, their market share remains relatively low (5% in 2015, 5% in 2019), probably due to the higher price of starting material N-type wafers [11] and the higher costs of fabrication (especially in the case of IBC and HJT). However, an increase of market share of N-type based crystalline silicon technologies is expected in the future [10] especially in applications with higher area-dependent costs. This increase can be more significant for PERT and TOPcon technologies that are closer to production methods broadly used in P-type technologies [12].

**Figure 6:**
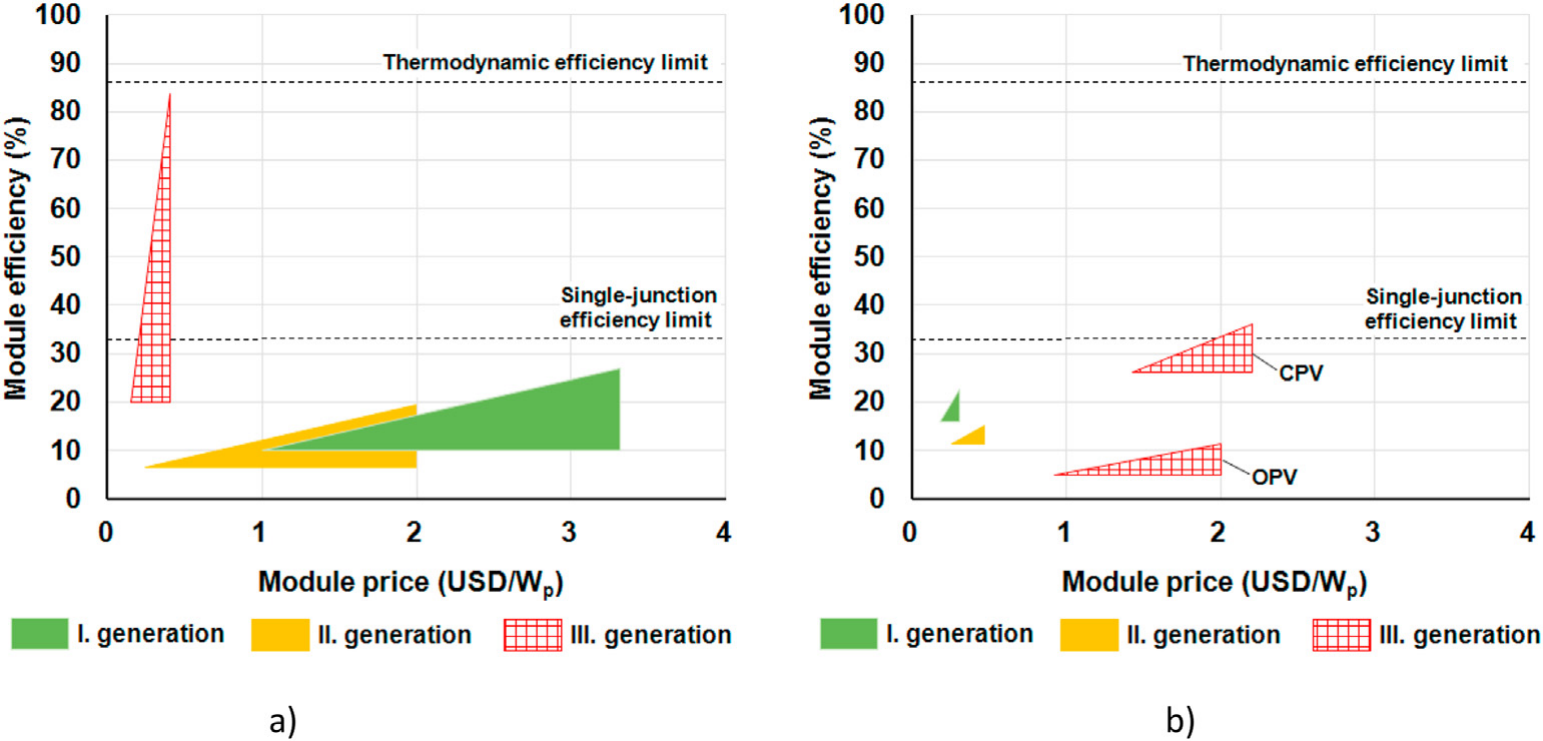
Efficiency and price of PV module generations a) assumption done in 2001 [13] b) reality in 2018.

From the viewpoint of R&D strategies, roadmaps are prepared on the basis of knowledge of materials and processes. In the field of photovoltaics, a general roadmap was presented in 2001 [13] that was based on analyses that crystalline silicon cells cannot be produced cheaper than 1 USD/W_p_ [14] and on the expectation of fast improvements in thin film and other emerging technologies. This roadmap divided materials and fabrication technologies of PV cells into three generations, as shown in Figure 6a. The first generation was represented by wafer-based crystalline silicon cells, relatively efficient, but expensive and therefore not too encouraging. The second generation, represented by single junction thin film cells, was a little less efficient, but much cheaper and therefore more suitable for large scale applications. The efficiency of the first and second generation cells cannot exceed the Shockley-Queisser limit for single absorber material device [15]. The term “third generation PV” was then used for devices with a potential efficiency above the Shockley-Queisser limit (tandem cells) and emerging technologies using new materials (DSSC, organic and polymeric solar cells, perovskite cells, quantum dot cells). Solution-processes were expected to bring a combination of high efficiency and low fabrication cost. This categorisation of three generations has been used frequently over the past 15 years.

Due to production improvements during this period, the price of semiconductor silicon decreased quickly from 500 USD/kg in 2007 to 55 USD/kg in 2010 and below 20 USD/kg since 2014 (the present price is about 10 USD/kg). Improvements in wafering, cell and module fabrication technologies brought the crystalline silicon to its leading position in PV module technologies depicting the generation scheme, as demonstrated in Figure 6b. Therefore, it is problematic to keep the three generation paradigm in its original form and is suggested [16] to replace it with the development of four generations of crystalline silicon technology dependent on the production process maturation.

## 3 PV technology development

As discussed above, photovoltaic components, especially photovoltaic modules, are required to have.

- Low price
- High efficiency
- Long service life
- No material supply constraints
- Prospects for further cost reduction

**Figure 7:**
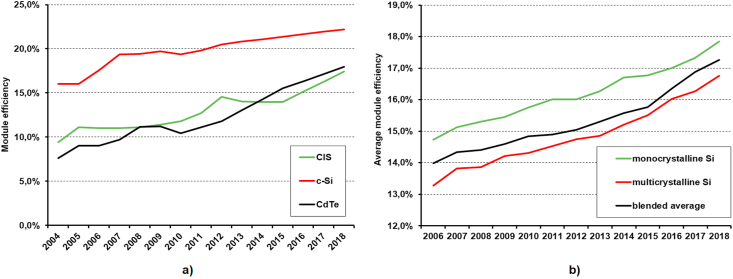
a) The development of the efficiency of the top products on the market. b) The development of the average efficiency of crystalline silicon modules (based on [17]).

At present, these requirements are best met by crystalline silicon modules. These modules currently have an efficiency of 16–22%. The trend of increasing the efficiency of mass-produced PV modules is demonstrated in Figure 7. Figure 7a shows the development of the efficiency of the top products on the market depending on the cell materials, Figure 7b shows the development of the average efficiency of crystalline silicon modules. There is currently a growing interest in changing from BSF cells to PERC cells with higher efficiency, from multicrystalline to monocrystalline starting wafer and also a growing interest in bifacial modules in which reflected radiation can be used to increase the output power.

The service life of the modules depends on the materials and technologies used in the lamination process, especially on the back cover material. When using plastic sheeting, the service life reaches 25 years, while when using glass sheeting, service life is longer and reaches 30–40 years [18]. The longer service life of glass/glass module is due to better resistance to higher temperatures, humidity and UV conditions; moreover, it has better mechanical stability, reducing the risk of microcracks during installation and operation. Despite the increased weight and price, glass/glass modules will be used to a greater extent mainly in connection with bifacial technology [10].

**Figure 8:**
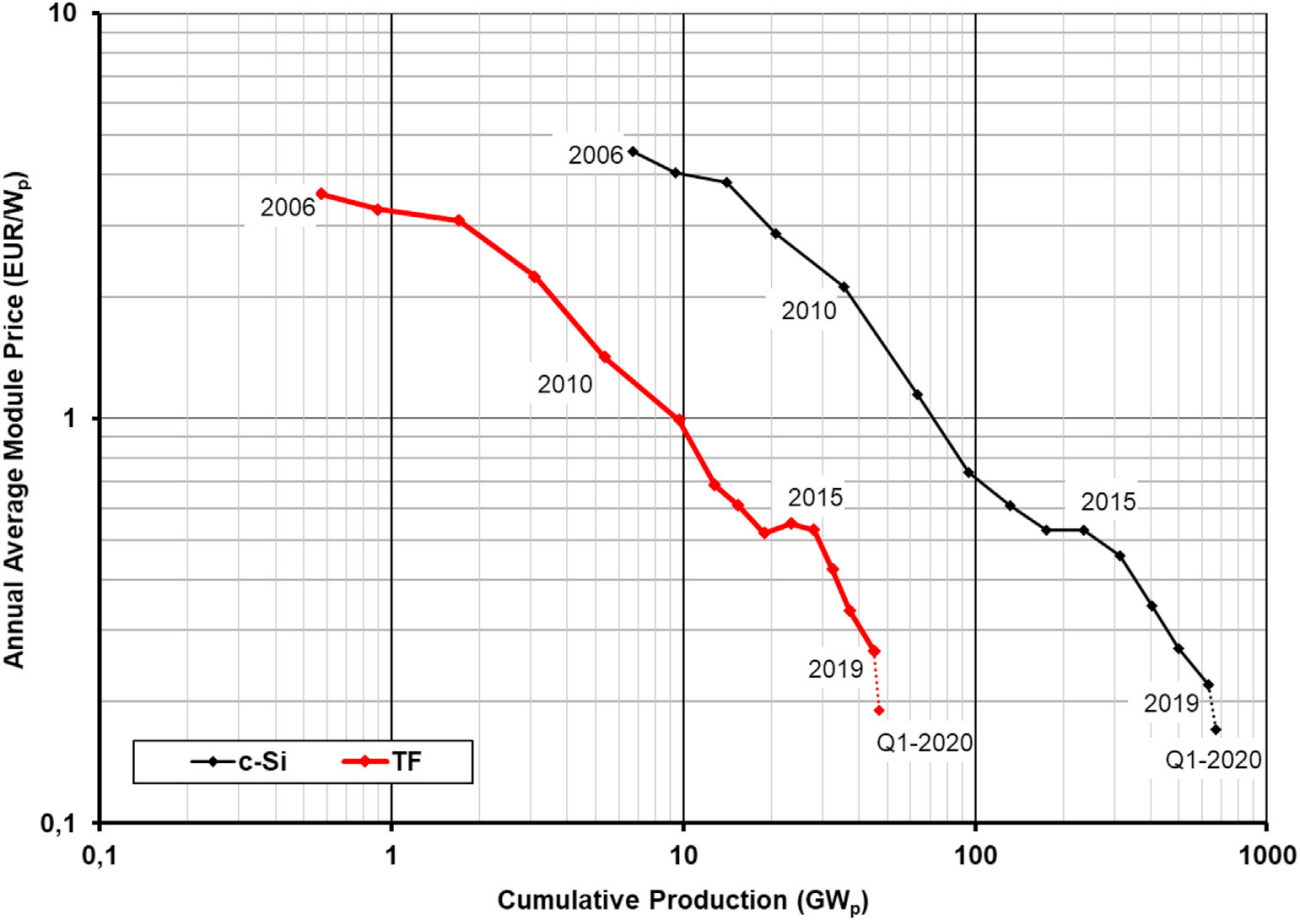
The development of module prices in dependence on the cumulative production [17, 19].

At the same time, the current cost of crystalline silicon modules is lower than the cost of modules from other materials due to the large-scale production of silicon feedstock, silicon ingots and wafers, silicon cells and modules. The PV silicon industry has an efficient supply chain, with high standardisation and other factors, including relatively low profit margins. The development of module prices is dependent on the total production volume (the so-called learning curve) as shown in Figure 8 [17]. Photovoltaic technologies, including silicon and thin film solar cells, have experienced unprecedented cost reductions among electricity-conversion technologies. The next cost decrease can be expected to be achieved through new technology, going to new processes (N-type material, bifacial, whatever it happens to be), or through increasing the level of existing operations by exerting greater control and organizing better operations or just increasing the throughput of the supply lines. Cost is still the weakness for the N-type as it has higher wafer material costs than P-type wafers and higher manufacturing costs.

In April 2020, the price of modules from multicrystalline Si was 0.160–0.290 USD/W_p_, (on average 0.177 USD/W_p_), the price of high efficiency monocrystalline Si modules was 0.185–0.380 USD/W_p_ (on average 0.200 USD/W_p_), the price of thin film modules was 0.200–0.320 USD/W_p_ (on average 0.221 USD/W_p_) [19]. A further decrease of the price of crystalline Si modules to the level of 0.150 USD/W_p_ can be expected before 2025 [20].

Currently, thin film technology modules are lagging behind crystalline silicon modules in both efficiency and cost, and have a somewhat shorter service life. The advantage of thin film modules is the smaller efficiency drop with temperature, which is advantageous for areas with high solar radiation intensity. Thin film technologies may also be used in building integrated PV applications and CIGS can have many applications as flexible PV modules. Even the efficiency of CdTe and CIGS modules increased from 10–13% to 14–16%. The proportion of thin-film modules as a share of total production is declining; currently it is about 5% of total production, with the largest decrease to be found in amorphous silicon modules production [17].

**Figure 9:**
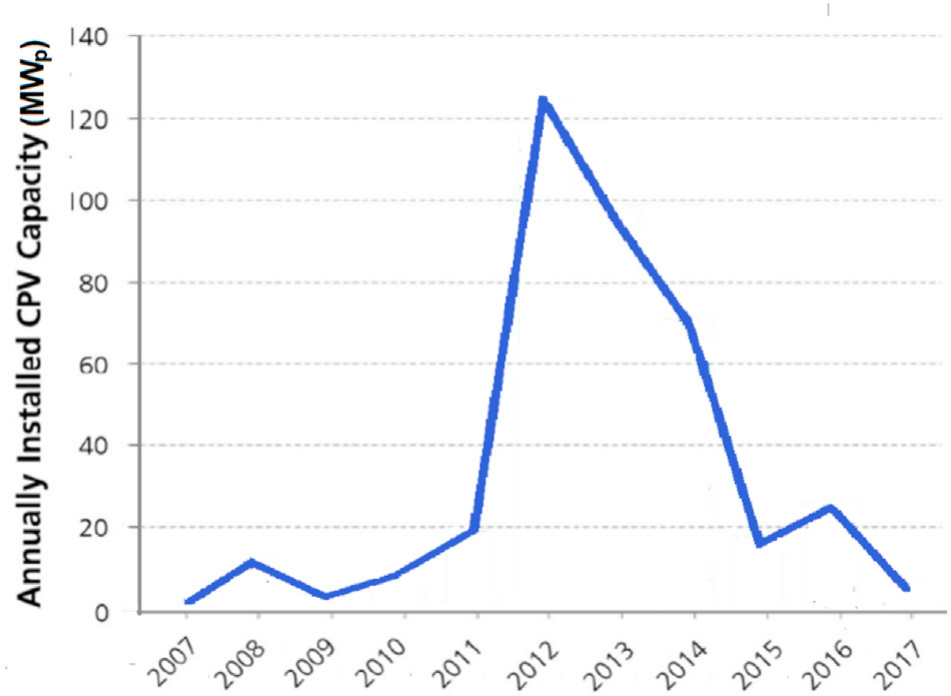
Annually installed CPV capacity development.

Although the technology of multi-junction high efficiency cells in concentrator systems (CPV) has the highest module efficiency, published data [17] shows that the market has not accepted it as demonstrated in Figure 9. The reasons are, on the one hand, some disadvantages of CPV systems (the utilization of direct irradiance component only, the need for tracking with sufficient accuracy and reliability, the need for cell cooling and lack of production technology standardization) and, on the other hand, the rapid decline in the prices of crystalline silicon modules. Market entry of new technologies (such as organic PV and quantum-dot PV) has been practically impossible due to poor cost competiveness in recent years [16].

From this technological family, the most promising are recently developed hybrid organic–inorganic metal halide perovskite. Perovskite solar cells have a great potential to become one of the leading technologies in the PV industry due to their high efficiency (about 20% on laboratory cell samples) and low manufacturing costs. There are two paths for further development. The first is fabrication of thin film perovskite cells on flexible substrates [21], the second may be tandems of crystalline silicon and thin film cells, where efficiency over 30% might be reached [22] by using the advantages of wafer-based technology. Crystalline silicone proven technology could serve as a very good bottom cell in tandem and in combination with a perovskite top cell, making a promising path for future development. A serious problem of perovskite structures was low stability. At present the stability has been improved and perovskite structures are nearing pilot line production, both flexible module roll-to-roll line [21] and the silicon – perovskite tandem [37]. Improvements in the quality and stability of perovskite modules are still the subject of intensive research and development [23]. Although some types of perovskite modules meet standard IEC 61215 testing (accelerated stress tests developed for silicon PV crystalline Si modules), the service life in field conditions is still not fully satisfactory [24, 25, 26]. Improvements in testing perovskite PV modules for stability are discussed in [27]. An extensive review on the evolution of perovskite solar cell development with an environmental impact and economic cost perspective has been carried out in [28]. Further improvements to cost and service life will be important for reaching competitiveness.

**Figure 10:**
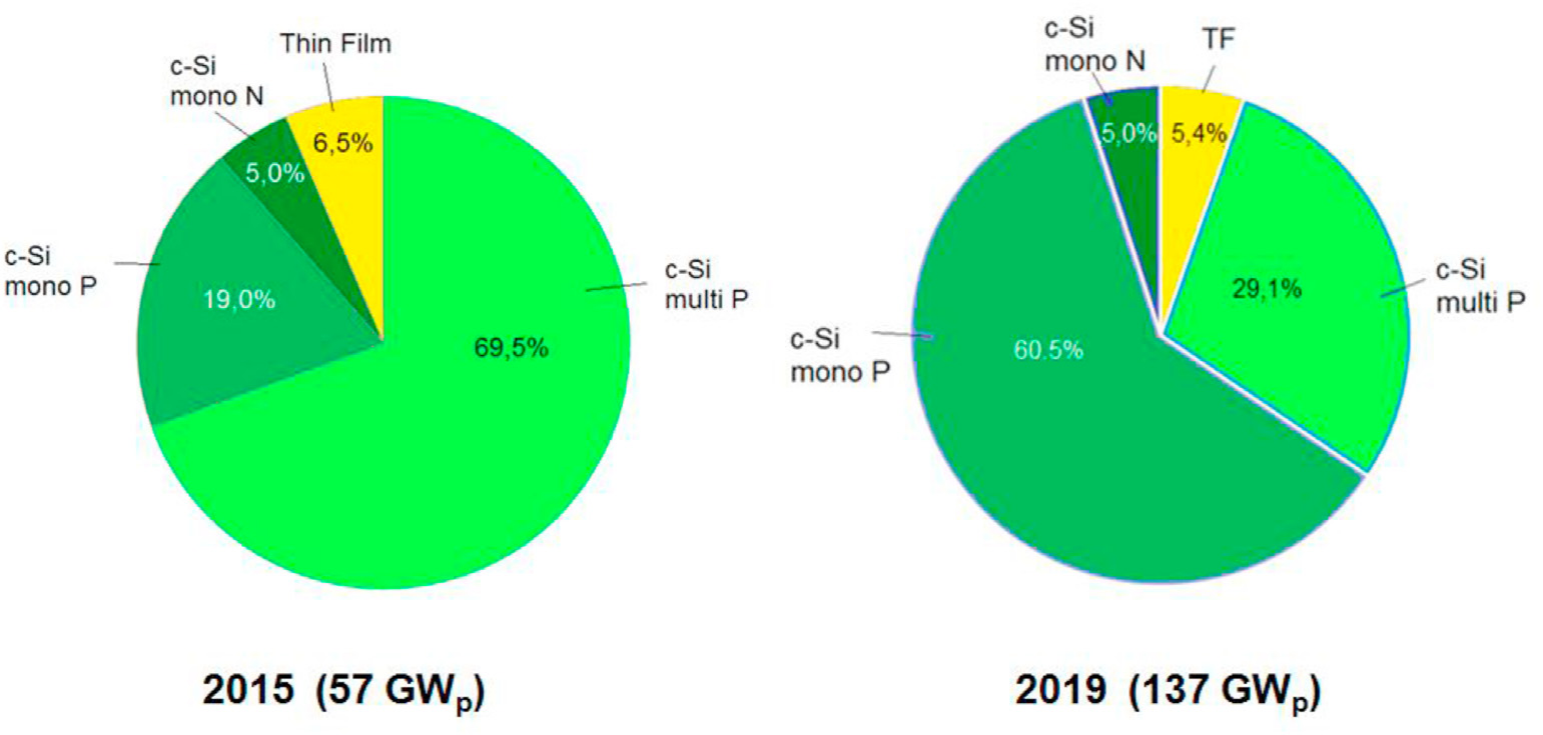
A comparison of market share by technology in 2015 and 2018.

A comparison of the share of individual technologies in total PV module production in 2015 and 2019 17] is shown in Figure 10.

## 4 Limits for present PV module technologies

**Figure 11:**
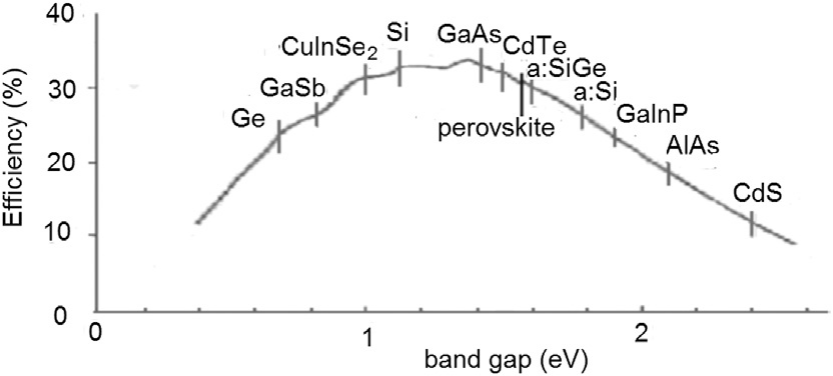
Maximum conversion efficiency in dependence on bandgap (spectrum AM 1.5).

For single material absorber semiconductor solar cells the maximum cell efficiency is limited by inter-band processes [15]. It depends on the material band gap and the irradiation spectra applied. For terrestrial use, the AM 1.5 spectrum is generally used for comparison of different cell parameters. The dependence of the efficiency physical limit on the material band-gap for the AM 1.5 solar spectra is shown in Figure 11. Due to losses connected with cell construction and fabrication, the operational efficiency is lower. The most advanced technology at this time is crystalline silicon technology where cell samples reached 93.2% of the physical limit, that is η = 29.43% [29]. Cells of efficiency of 85% of the physical limit are already in series production. With other materials the ratio between the real technology and the ultimate efficiencies is lower. Using c-Si – perovskite tandem cells could increase the cell efficiency above the 30% threshold, which would result in module efficiency over 25%.

**Figure 12:**
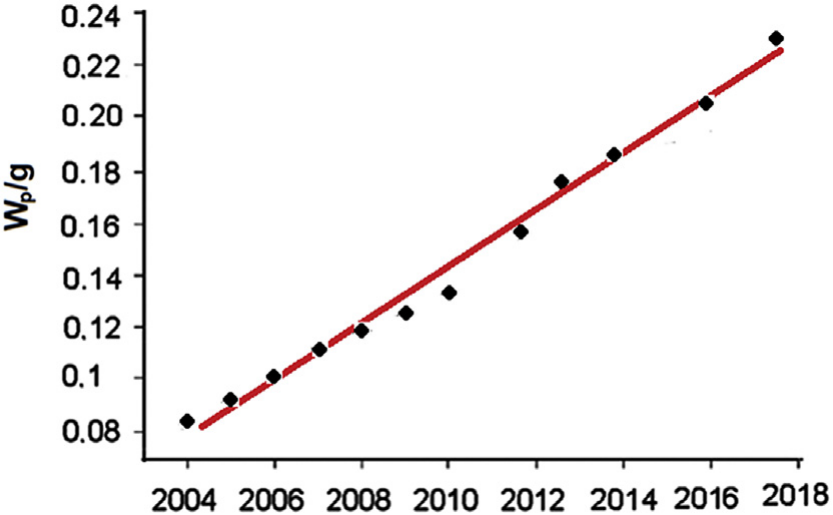
Development of peak power produced from 1 g of c-Si.

Some additional losses can be also attributed to module fabrication, such as the transparency and reflectivity of top covering layer (e.g. glass), etc., keeping the module efficiency below the cell efficiency. An increase in module power output is possible to obtain also using the half-cut cell technology or the shingling design [8]. All of these step-by-step improvements result from more effective use of starting material, as demonstrated in Figure 12.

From the viewpoint of future deployment in the terawatt era, there may be some problems with the limited resources of In, Te and Se. Resource limits are discussed in [30]. For both CdTe and CIGS modules, the maximum production limit was stated as 20 GW_p_ a year, which is far below expected demand for several hundred GW_p_ a year in future. In the case of crystalline silicon modules, all of the materials are abundant and some limits are connected only with Ag resources which limits possible future production to about 2 TW_p_ a year.

A decrease in LCOE produced by PV systems can be realised not only by decreasing costs both PV modules and BOS components. The technological change resulting in a higher power PV modules (with a power output over 400 W_p_) will also lower BOS costs.

As follows from (2) and (3), an increase of DC to AC conversion efficiency *η*_*c*_ can also play a significant role in the LCOE decrease along with increasing yearly irradiance *H*_*sy*_ on the module surface by solar tracking. Another way to increase the irradiation of the module surface is to use bifacial modules, especially in places with high surface reflectivity. Because of this, bifacial modules have a great potential to become key in utility scale photovoltaic power plants.

Another current trend in PV power stations is increasing the string DC voltage to 1500 V. At this higher voltage level, it is possible to realize longer strings and reduce the number of inverters as well as the cost of cables and structures, thus reducing installation and maintenance costs. However, it increases demands on the dielectric strength of modules and higher internal electric fields may be reflected in PID -like defects, and also on the personnel's qualifications (legislative restrictions over 1 000 V).

**Figure 13:**
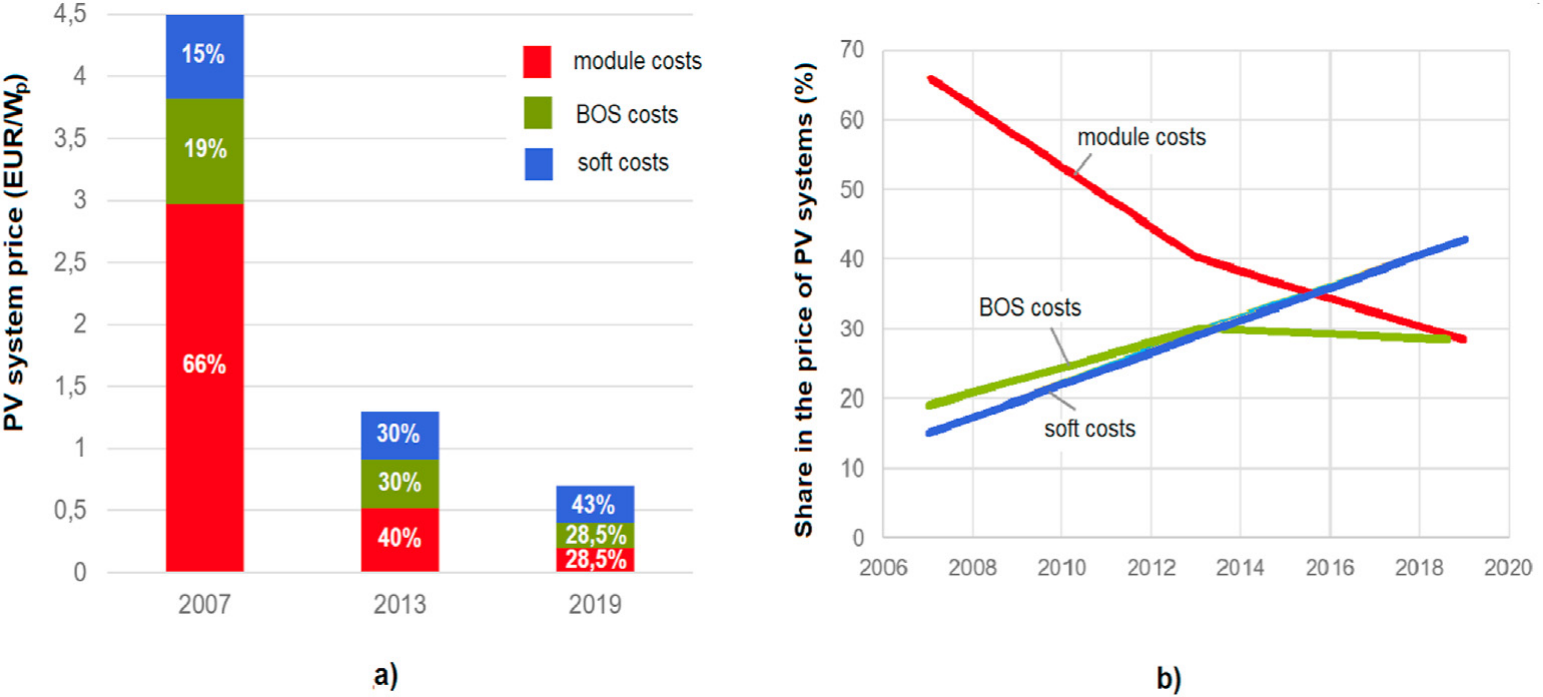
a) Utility-scale PV systems price and price structure development. b) The share in the price of utility-scale PV systems.

Investment costs consist not only of the costs of modules and the BOS components (i.e., inverters, constructions, installations, monitoring, etc.), but also of the so-called “soft costs” (project, fees, margins, financing, etc.) [31]. The investment cost structure development for utility-scale PV systems [32] is shown in Figure 13a. With the development of technology, the cost of BOS modules and components decreases rapidly, unlike "soft costs". In this way, the share of "soft costs" in the investment costs increases, as shown in Figure 13b. In the case of residential-scale PV systems, the share of "soft costs" can be even higher, up to 60% [31]. Soft costs are associated with legislation and vary considerably from country to country, which somewhat complicates any analysis.

Although the costs of modules cease to be the largest part of investment costs, the technical and economic parameters of modules remain one of the key factors in the further development of photovoltaics.

## 5 Conclusions

In the present developments of photovoltaics, wafer-based crystalline silicon technologies have the role of the work-horse of present PV power generation, representing nearly 95% of total module production thanks to their high efficiency, low cost and a long service time. Further technological improvements have to be implemented to support the decrease in LCOE of energy generated by PV systems that may play an important role in increasing the role of renewable energy in the future [33]. In this respect, only technologies that meet the criteria for service life (more than 15 years), efficiency (more than 14%) and cost (less than 0.3 USD/W_p_) and that are not significantly constrained in resources can represent a major technological trend in energy applications. Currently, these assumptions have modules based on crystalline Si technology that would remain the work-horse position for a minimum of the next five years [34]. Then it might be possible to assume a more significant application of the technology of perovskite modules, if their operational reliability is successfully solved.

Until now, the development of the photovoltaic industry has always been dependent on subsidies. Due to the lowering of the purchase price, increasing the efficiency and lifetime of the photovoltaic systems, photovoltaics have become competitive in terms of comparing the LCOE with other energy sources [35] in a substantial part of the world.

## Declarations

### Author contribution statement

All authors listed have significantly contributed to the development and the writing of this article.

### Funding statement

This work was supported by project TACR No. TK03020144 and by the Czech Ministry of Education, Youth and Sports Project CZ.02.1.01/0.0/0.0/15_003/0000464 – “Centre of Advanced Photovoltaics”.

### Data availability statement

No data was used for the research described in the article.

### Declaration of interests statement

The authors declare no conflict of interest.

### Additional information

No additional information is available for this paper.
